# Implementation of a surgical site infection prevention bundle: Patient adherence and experience

**DOI:** 10.1017/ash.2021.214

**Published:** 2021-12-10

**Authors:** Stacey Hockett Sherlock, Daniel Suh, Eli Perencevich, Heather Schacht Reisinger, Judy Streit, Amy Frank, Gosia Clore, Madeline Ohl, Dina Speicher, Loreen Herwaldt, Marin L. Schweizer

**Affiliations:** 1 Iowa City VA Health Care System, Iowa City, Iowa; 2 Department of Internal Medicine, University of Iowa Carver College of Medicine, Iowa City, Iowa; 3 Department of Epidemiology, University of Iowa College of Public Health, Iowa City, Iowa

## Abstract

We evaluated barriers and facilitators to patient adherence with a bundled intervention including chlorhexidine gluconate (CHG) bathing and decolonizing *Staphylococcus aureus* nasal carriers in a real-world setting. Survey data identified 85.5% adherence with home use of CHG as directed and 52.9% adherence with home use of mupirocin as directed.

Prior studies have demonstrated that chlorhexidine gluconate (CHG) bathing and decolonizing *Staphylococcus aureus* nasal carriers prevents surgical site infection (SSI) after total joint arthroplasty (TJA).^1,2^ However, one study reported that the effect was only beneficial among patients who were fully adherent with these interventions.^1^ We assessed patient adherence with mupirocin and CHG and characterized factors associated with improved patient adherence.

## Methods

In April 2012, our hospital implemented a bundled approach to prevent *S. aureus* SSI among patients undergoing TJA as the standard of care. Patients were tested for *S. aureus* nasal carriage during their preoperative clinic visits within 30 days before scheduled TJA. Polymerase chain reaction (PCR, Cepheid XPert SA Nasal Complete) was used to identify both methicillin-susceptible and methicillin-resistant *S. aureus*. Results were reported to the preoperative clinic on the same day as the visit. Nurses educated patients on use of CHG and mupirocin. Patients who were *S. aureus* carriers were prescribed 2% nasal mupirocin ointment to self-apply twice daily and CHG soap (Hibiclens 4%, Hibiclens, Norcross, GA) to use daily for the 5 days before surgery. Patients who were not *S. aureus* carriers were prescribed CHG soap to use the day before and the morning of surgery. Prescriptions were sent to the outpatient pharmacy for the patient to obtain before leaving the hospital.

On the morning after TJA, a researcher obtained verbal consent from the patient and their nurse to administer an oral survey. Surveys consisted of 31 items including patient demographics and use of mupirocin and CHG. Surveys were administered between July 2018 to January 2020. Patients were categorized as *S. aureus* carriers if they recalled receiving mupirocin prescriptions, and this was confirmed through chart review. The VA Central Institutional Review Board and the Iowa City Research and Development Committee approved this study.

Study data were managed using Research Electronic Data Capture (REDCap) tools. The Fisher exact test was used to compare categorial variables, and the Wilcoxon 2-sample test was used to compare continuous variables. For the statistical analyses, we used SAS Enterprise Guide version 8.2 software (SAS Institute, Carey, NC).

## Results

Overall, 286 TJA were performed, and 77 patients (26.9%) completed the survey. In total, 17 patients (22.1%) were categorized as *S. aureus* carriers; of these, 2 had a previous positive carrier result and reported using mupirocin. Furthermore, 60 patients (77.9%) reported full adherence based on their *S. aureus* carriage status.

**Fig. 1.**
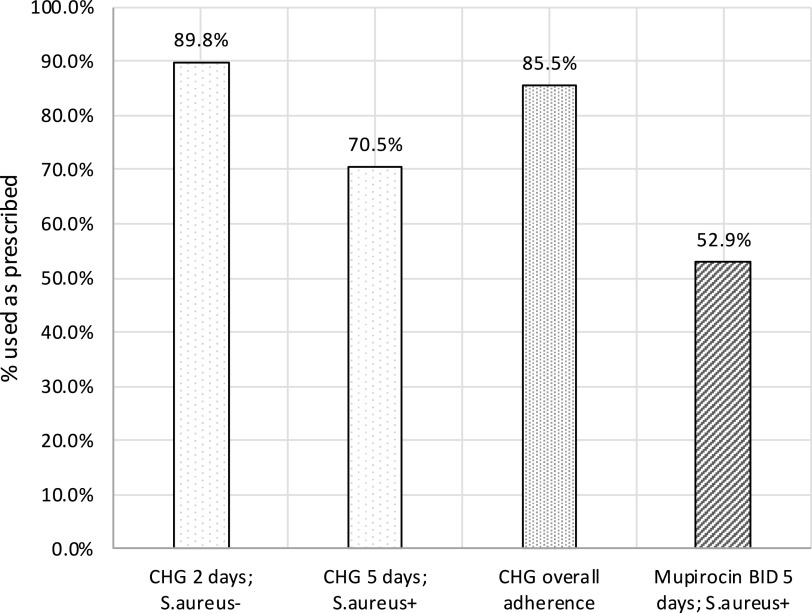
Adherence with chlorhexidine gluconate (CHG) bathing and mupirocin by methicillin-resistant *S. aureus* (MRSA) and methicillin-susceptible *S. aureus* (MSSA) carrier status.

All but 1 patient recalled receiving CHG soap (98.7%). Among the 76 patients who recalled receiving CHG soap, 65 patients (85.5%) used CHG as prescribed (Fig. 1). Also, 59 patients who were not *S. aureus* carriers recalled receiving CHG soap, and 53 (89.8%) used CHG for 2 days as prescribed. Among the 17 patients who were *S. aureus* carriers, 12 (70.5%) were fully adherent with the 5-day regimen. In total, 94.7% recalled receiving instructions on CHG use. Patients who received CHG from the hospital pharmacy were significantly more likely to be fully adherent with CHG bathing compared with patients who received CHG through the mail or a different method. Patients who stated that they did not have a barrier to using CHG were also more likely to be fully adherent with CHG (Table 1). Barriers to patient adherence with CHG bathing included forgetfulness (n = 2), difficulty with daily bathing (n = 2), having a side effect (n = 1), and not having enough product for days needed (n = 1). Also, 8 patients (10.5%) reported CHG side effects, including burning (n = 3) or tight, itchy, or dry skin (n = 4) or allergic reaction (n = 1). Furthermore, 75 (98.6%) patients stated that they were willing to use CHG before a future surgery.

**Table 1.** Characteristics of Surveyed Patients by Chlorhexidine Gluconate (CHG) and Mupirocin Adherence

Characteristic	Fully Adherent with CHG (n=65), No. (%)	Not Fully Adherent with CHG (n=11), No. (%)	Total (n=76), No. (%)	Fully Adherent with Mupirocin (n=9), No. (%)	Not Fully Adherent with Mupirocin (n=8), No. (%)	Total (n=17), No. (%)
Prior history of *Staphylococcus* infection among self or friend	24 (36.9)	4 (36.3)	28 (36.8)	3 (33.3)	5 (62.5)	8 (47.1)
**Received product from**						
Hospital Pharmacy	58 (89.2)	7 (63.6)	65 (85.5)	9 (100)	5 (62.5)	14 (82.4)
Mail	3 (4.6)	2 (18.2)	5 (6.6)	0	2 (25.0)	2 (11.8)
Other	4 (6.2)	2 (18.2)	6 (7.9)	0	1 (12.5)	1 (5.9)
Remembered receiving instructions for use	63 (96.9)	9 (81.8)	72 (94.7)	9 (100)	8 (100)	17 (100)
Reported ≥1 barrier to use	2 (3.1)	4 (36.4)	6 (7.9)	0	1 (12.5)	1 (5.9)
Experienced side effect(s)	6 (9.2)	2 (18.2)	8 (10.5)	2 (22.2)	3 (37.5)	5 (29.4)
Age, median y (IQR)	69 (60–72)	70 (67–72)	69 (60–72)	68 (63–70)	74 (69–77)	70 (67–74)
More than high school degree	40 (61.5)	8 (72.7)	48 (63.2)	7 (77.8)	5 (62.5)	12 (71.6)
Annual income >$25,000^a^	50 (79.4)	8 (72.7)	58 (76.3)	8 (88.9)	3 (37.5)	11 (64.7)
Willing to use again	65 (100)	10 (90.1)	75 (98.6)	9 (100)	8 (100)	17 (100)

Note. IQR, interquartile range.

a 1 person preferred not to answer in mupirocin analysis; 3 people preferred not to answer in CHG analysis.

Of the 17 patients who recalled receiving a mupirocin prescription, 9 (52.9%) used mupirocin twice a day for 5 days. All 17 patients remembered receiving instructions on how to use mupirocin. Younger patients and patients with incomes >$25,000 per year were significantly more likely to be fully adherent with mupirocin (Table 1). Reported side effects associated with mupirocin were stinging, itching or dryness (n = 2), unpleasant taste (n = 2), runny or stuffy nose (n = 3), and other side effect (n = 1). Forgetfulness was the only reported barrier to mupirocin use (n = 1). All patients who used mupirocin stated they were willing to use mupirocin before a future surgery.

Facilitators of patient adherence with mupirocin and CHG included high facility adherence with *S. aureus* screening (98.7% patients reported) and access to prescribed medications (98.7% patients received). Patients who had someone to help them with personal care or remind them to use medications were significantly more likely to be adherent with CHG.

Patients were asked whether they would prefer to have a nurse in hospital apply a liquid medication (eg, povidone iodine) to their nares on the day of surgery or self-apply an ointment to their own nares for 5 days before surgery at home (as they did if using mupirocin). Among the 17 patients who received a mupirocin prescription, 14 (82.4%) preferred to self-apply ointment and 3 (17.7%) had no preference.

## Discussion

This evidence-based intervention is recommended by the World Health Organization^3^ and was standard of care at this hospital for 6 years before our survey. In our study, patients remembered being educated about how to use mupirocin and CHG, and most stated willingness to use these products for a future surgery. However, 86% of patients were fully adherent with CHG bathing and only 53% were fully adherent with mupirocin. A previous survey of a decolonization bundle (mupirocin, CHG, and mouthwash) revealed that 71% of patients reported good compliance.^4^ Thus, hospitals implementing a decolonization bundle should consider interventions to overcome barriers to adherence.

Factors associated with full adherence included younger age and receiving the products through the hospital pharmacy. To improve adherence, hospitals may need to adapt instructions for older adults and for patients receiving their medications by mail. Strategies for adhering to short-term medication regimens have not been well studied.^5^ Forgetfulness was a barrier that may be remedied by a patient reminder in the week before surgery^6,7^ or by instructing patients to use contextual cues.^5^

To understand patient preferences, we asked patients if they would prefer to self-apply at home for 5 days (as they did if mupirocin was used), or for a nurse to apply on day of surgery (eg, povidone iodine). Every patient who received mupirocin either preferred self-application or had no preference, and none preferred nurse application. Mupirocin adherence was low, but patients reported few barriers, suggesting that the patients believed that they used mupirocin correctly and that their nonadherence was unintended.^8^ Thus, hospitals need to assist patients in using the medication correctly, or replace mupirocin with a day-of-surgery decolonizing agent^9^ that does not rely on patient adherence.

This study had several limitations. All data were self-reported, and we had a small sample size of *S. aureus* carriers. We did not assess whether adherence reduced the SSI rate. However, our prior study found a significant association between adherence with these interventions and a reduction in *S. aureus* SSIs.^1^

In this study, we evaluated the real-world acceptability and adherence with interventions in which the preoperative orthopedic clinic nurses, laboratory, and pharmacy worked together to ensure successful implementation. These results may not be generalizable to patients having emergent surgery, patients not seen in preoperative clinics, and lower-resource settings.^10^ Future studies should evaluate interventions such as patient reminders or simplifying the process with other intranasal decolonizing agents, to increase patient adherence.
